# Chimeric Free Fibula Osteocutaneus Flap and Massive Allograft for Refractory Post-traumatic Osteomyelitis Femur Defect: A Case Report

**DOI:** 10.5704/MOJ.2503.017

**Published:** 2025-03

**Authors:** HY Lam, AS Halim, WA Wan-Sulaiman

**Affiliations:** Department of Reconstruction Science Unit, Universiti Sains Malaysia, Kubang Kerian, Malaysia

**Keywords:** chimeric free fibula flap, allograft, refractory osteomyelitis, femur

## Abstract

Surgical management of femur osteomyelitis remains challenging. The burden of this chronic disease invariably results in composite bony and soft tissue defects that can interfere with bony stability. Therefore, reconstructive surgery is integral to functional limb salvage and limb preservatives. To the best of our knowledge, we are the first to report the limb salvaging method and important planning considerations for a case of chronic refractory osteomyelitis. We presented a case of a 31-year-old female with chronic post-traumatic osteomyelitis of the right femur. This intractable disease results in frequent remission of infection and non-union of the midshaft fracture. Surgical management with the implant, external fixation, and cement spacer failed due to infection. This rendered vascularised bone graft with massive allograft the only option. We described the anatomical aberrant during the harvest of free fibula flap and modified chimeric fibula flap to overcome the soft tissue defect complicated with severe fibrotic tissue with a background of an obese patient. She had undergone emergency hematoma evacuation 20 hours after the surgery. Otherwise, the flap survived well, and the patient started to have partial weight bearing. Chimeric fibula osteocutaneous free flap is a useful armament to provide a complex 3-dimensional spatial arrangement in a case of chronic osteomyelitis with huge bony and soft tissue defects.

## Introduction

Antibiotics and surgical debridement are the most important armaments to treat chronic osteomyelitis. However, treatment failure or relapse remains at 20% in a year^1^.

Chronic refractory osteomyelitis (CROM), which is resistant to traditional surgery and antibiotics, has a considerable morbidity and mortality rate in addition to a detrimental effect on patient quality of life due to pain and impaired function.

## Case Report

**Fig. 1:**
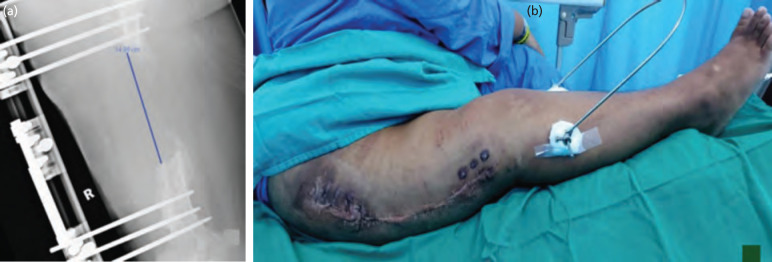
(a) The defect over right femur measuring about 14.95cm. (b) Pre-operative wound of right thigh showed significant scarring secondary to chronic osteomyelitis.

**Fig. 2:**
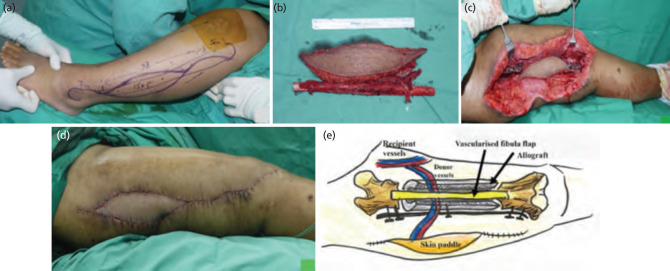
(a) Harvesting vascularised free fibula with the osteocutaneous fibula flap with 15cm of the osseous component. (b) Chimeric - free fibula flap. (c) Inset of the chimeric free fibula flap. (d) The vascularised fibula flap included a whole allograft joined to the fibula flap as a biologically vascularised unit.

A 31-year-old lady presented with a closed fracture of the right midshaft femur, where she had open reduction and plating of the right femur. Two months post-operatively, she had painful right thigh swelling. She was treated with systemic antibiotics for the surgical site infection and implant failure. She has the infected implant removed. After another cycle of wound debridement, a monorail external fixation was applied. The erythrocyte sedimentation rate, and C-reactive protein were abnormal. She had multiple surgical wound debridement and sequestomy to eradicate the necrotic tissue and bone one year after the trauma. After the wound bed and ESR were optimised, an antibiotic cement spacer was employed to provide temporary sterility and stabilisation of the osseous defect. However, she presented with pus discharge from the surgical pin site. The orthopaedic team had no choice but to remove the cement spacer and performed extensive debridement to remove nonviable bone and tissue. This resulted in a huge femur defect of 15cm in length with unhealthy soft tissue in the lateral aspect of the thigh (Fig. 1a). Limb-sparing surgery employed an osteocutaneous fibula-free flap combined with a massive allograft to provide healthy bone and soft tissue (Fig. 2a).

For surgical technique, after adequate debridement and excision of the scar, this left a large soft tissue defect over the lateral aspect of the right thigh. We harvested an osteocutaneous fibula flap with 15cm of the osseous component, based on a single myocutaneous peroneal perforator as there was absence of a septocutaneous perforator. Flap inset was done via medial approach as (i) it was closer to the profunda femoral artery for anastomosis and (ii) scarring over the lateral thigh due to previous surgical debridement posed risks of dissection for flap inset (Fig. 1b). On the other hand, it was difficult to establish the optimum skin paddle axis when utilising the skin paddle in the conventional free fibular osteocutaneous flap design to resurface the soft tissue defect at the lateral aspect of the thigh. Additionally, we needed to bridge 15cm between the skin paddle and the main perforator from the medial approach due to obesity. Therefore, we employed the chimeric-free fibular osteocutaneous flap, where the perforators to the skin paddle were separated from the localised bone component (Fig. 2b). The skin paddle of the fibula osteoseptocutaneous flap is typically well vascularised by septocutaneous perforators from the peroneal artery. However, in 5% to 10% of lower limbs, these perforators may be absent, as observed in our case^2^. Consequently, the muscular perforators supplied by the posterior tibial vessels were harvested as the primary pedicle, situated near the posterior septum. This approach necessitated dissection through the anterior aspect of the soleus muscle. The intricate 3-dimensional thigh anatomy could be recreated using this design, which allowed different tissue components to be spatially placed (Fig. 2c). The vascularised fibula bone was inserted in the intramedullary position of the massive allograft and stabilised with a locking plate and screw (Fig 2d and 2e). The vascularised fibula flap included a whole allograft joined to the fibula flap as a biologically vascularised unit. The skin paddle was valuable for postoperative monitoring and soft tissue restoration.

**Fig. 3:**
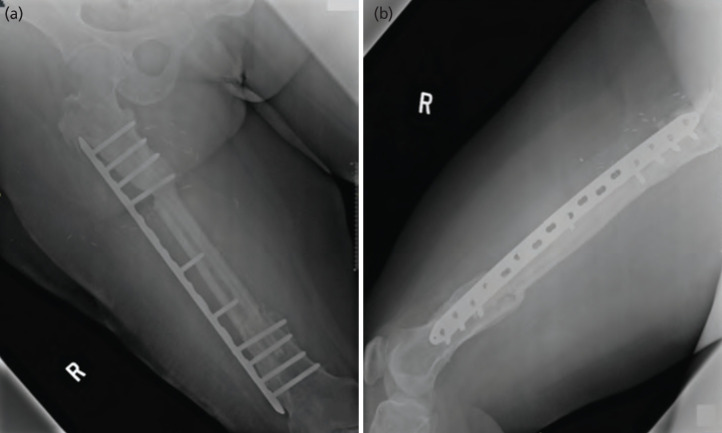
AP and lateral view of the right femur two years after the reconstruction shows good bony union with resolution of chronic osteomyelitis.

The patient underwent emergency exploration to evacuate the hematoma that compromised the skin paddle perforator during post-operative day 1. The flap survived well, and she was discharged after two weeks of hospital stay. Follow-up two years after the surgery shows good recovery of the flap and good bone union seen from the radiograph (Fig. 3).

## Discussion

Osteomyelitis may develop due to contamination of open fractures or open repair of closed fractures. The clinical presentation of post-traumatic osteomyelitis results in poor wound healing and fracture union. Fever, wound drainage, warmth, pain, and erythema may occur. Established osteomyelitis often warrants combined medical and surgical therapy. Because antibiotics penetrate dead or injured bone and infected fluid collections poorly, surgical debridement is a cornerstone of therapy when these are present. Ten to Thirty percent (10% to 30%) of acute cases progress to chronic conditions^3^.

Chronic bone infections that did not improve with standard medical treatment and antibiotics cause considerable morbidity, mortality, and poor quality of life for patients attributable to pain and impaired function. Surgical debridement invariably entails bone loss and surrounding soft tissue defects. In case of severe infection, history of recurrence, and antibiotic resistance, major limb amputation is the last option when other interventions fail^4^. With the evolution of microsurgery and implant devices, limb salvage outperforms amputation. Trung *et al* have reported total femur replacement to treat chronic refractory osteomyelitis^4^. Post-operative infection is also significant for individuals with total femur replacement (TFR) and associated infections. One in six patients experienced re-infection three months after surgery when TFR is used to treat infection surrounding a prosthesis. According to Corona's 2018 study, 17.2% of patients still had post-operative infections^5^.

Germane to this discussion, reconstruction of resistant chronic osteomyelitis limb poses significant challenges. The contentious issue is the bone and soft tissue defect after multiple surgical excisions, biological factors for fracture healing because of inadequate bone stock and disuse osteopenia, and stable fixation The Capanna approach described a single-stage reconstruction for femur defect, which combines allograft with vascularised bone graft designed to circumvent the problem, as mentioned earlier. The vascularised fibula bone supplies the osteoinductive, osteoconductive, and osteoprogenitor components required for a successful bony union. The allograft is utilised with the fibula to overcome the fibula's width discrepancy with the femur and enhance mechanical axial load stability. Subsequently, the modified Capanna technique highlights the advantages of the cutaneous component of free fibula osteocutaneous flap in combination with graft reconstruction. After removing scar tissue, the skin paddle provided healthy soft tissue to adjacent skin. In addition, the skin paddle is a useful parameter for clinical monitoring of the free flap post-operatively. The availability of massive allograft-facilitated extensive limb reconstruction is laudable. However, infection, non-union, and allograft fracture have inherent disadvantages. A free vascularised fibula enhanced host-allograft junction union. However, it has limited length, strength, and stability to support the mechanical axial load. Thus, the advantages of a stable bone stock from an allograft and the biological potentials of a free vascularised fibula flap form the perfect biological reconstruction. This biological limb reconstruction offers a good long-term outcome with imperceptible complication rates.

In our case, limb-sparing surgery with modified Cappana's technique is an excellent option to provide a complete biological vascularised unit with skeletal stability. Modification with chimeric osteocutaneous free fibula flap offers a unique configuration where the skin paddle and osteal flap can be spatially arranged to accommodate the complex geometry arrangement in the thigh. Existing literature advocated the good outcome of chimeric-free fibula flap in head and neck reconstruction. The chimeric flap can provide the multiple tissue elements necessary for complex reconstruction from a single donor site while simplifying pedicle management. Moreover, chimeric flaps can incorporate several independent tissue components without the added morbidity of multiple donor sites. However, each flap component comes with risks of vessel kinking. The substantial debridement in our case resulted in a hematoma to compress on the perforator to skin paddle. The problem had been successfully reversed by early discovery, prompt exploration, and the use of the doppler and skin colour changes. Proper fluid drainage by Radivac drain and positioning of the skin paddle is important to avoid vascular compromise.

We initially anticipated the lengthy surgery for dissection and flap inset due to the thickness of subcutaneous fat in this obese patient. Besides that, complications of microsurgery for the obese population are not uncommon. The average intra-operative ischemia time of a free vascularised fibular flap was longer in obese patients; this finding may be explained by the more challenging surgical access to neurovascular systems and the dissection of the donor and recipient areas. These patients may have higher rates of flap loss and donor site complications than their normal weight counterparts, as well as an increased risk of nosocomial and surgical site infections. This chimeric flap accomplishes complex 3-dimensional reconstruction with a single donor site and vascular pedicle. This reduced the timing to harvest two free flaps and less donor site morbidity. The chimeric free fibula flap allows for single-stage reconstructions, reducing the need for multiple surgical procedures. This is particularly advantageous in limb-preserving surgeries, where timely intervention is critical for patient recovery and rehabilitation. The skin paddle from the vascularised bone graft helps distribute tension evenly across the limb, contributing to better healing outcomes. Our patient was discharged well two weeks after the hematoma evacuation.

The reconstruction paradigm has changed from a climbing ladder approach to providing the most precise floor by elevator. Limb-sparing surgery in a single stage is the key to employing the elevator technique successfully. Chimeric osteocutaneous free fibula with large allografts offers an optimum configuration to local soft tissue defects while maintaining bone integrity, particularly in the case of persistent refractory osteomyelitis.

## References

[1] Spellberg B, Lipsky BA (2012). Systemic antibiotic therapy for chronic osteomyelitis in adults.. Clin Infect Dis..

[2] Schmitt SK (2017). Osteomyelitis.. Infect Dis Clin North Am..

[3] Wong CH, Tan BK, Wei FC, Song C (2007). Use of the soleus musculocutaneous perforator for skin paddle salvage of the fibula osteoseptocutaneous flap: anatomical study and clinical confirmation.. Plast Reconstr Surg..

[4] Trung DT, Dinh HN, Le NT, Luong LH, Nguyen TT (2021). Total femur replacement in a patient with chronic persistence osteomyelitis - A case report.. Int J Surg Case Rep..

[5] Corona PS, Vicente M, Lalanza M, Amat C, Carrera L (2018). Use of modular megaprosthesis in managing chronic end-stage periprosthetic hip and knee infections: Is there an increase in relapse rate?. Eur J Orthop Surg Traumatol..

